# Large Left Atrial Thrombus in a Patient With Severe Mitral Stenosis and Atrial Fibrillation Despite Anticoagulant Therapy: A Case Report

**DOI:** 10.7759/cureus.52634

**Published:** 2024-01-20

**Authors:** Silvio Nocco, Laura Concas, Marco Fei

**Affiliations:** 1 Department of Cardiology, Sirai Hospital, Carbonia, ITA

**Keywords:** cardiac mass, echocardiography, left atrial thrombus, left atrial dilatation, mitral stenosis

## Abstract

This case report highlights a patient with atrial fibrillation, severe mitral stenosis, and left atrial dilatation who developed a large thrombus, despite being on anticoagulant therapy. The complexity of thrombus formation in patients with multiple risk factors is described, emphasising the need for regular echocardiographic assessments to detect and monitor thrombi, even in patients undergoing anticoagulant treatment. The interplay between atrial fibrillation, mitral stenosis, and left atrial dilatation contributes to thrombus formation, requiring a multidisciplinary approach to the management of these patients. Further research is needed to improve our understanding of the optimal treatment strategies for such cases. Timely identification and intervention are critical to mitigate the risk of thromboembolic complications in these high-risk patients.

## Introduction

Patients with atrial fibrillation, severe mitral stenosis, and left atrial dilatation have a higher risk of developing left atrial thrombosis than those with atrial fibrillation alone [1]. While atrial fibrillation-related thrombosis is found in the left atrial appendage under normal circumstances, it is not uncommon for thrombi to form directly in the main chamber of the left atrium, even without appendage involvement, in the aforementioned patients [2]. The risk of thromboembolism increases with left atrial enlargement, regardless of anticoagulant therapy [3].

## Case presentation

An 82-year-old female patient was admitted to the emergency room following a syncopal episode and dehydration. For the past six months, she had experienced drowsiness and a worsening of her general condition. The patient had a history of mitral stenosis, which was corrected by valvuloplasty at the age of 70. However, approximately five years earlier, the valve condition recurred, and an echocardiographic evaluation revealed severe mitral stenosis. Due to the patient's comorbidities and age, cardiothoracic surgeons deemed her inoperable due to high risk. Additionally, she was diagnosed with type II diabetes mellitus, high blood pressure, and mild senile dementia.

The patient had received the following therapy prior to admission: warfarin, maintaining an INR therapeutic range between 2 and 3; furosemide, 150 mg daily; bisoprolol, 10 mg daily; digoxin, 0.0625 mg daily; candesartan, 16 mg daily; esomeprazole, 40 mg daily; mirtazapine, 15 mg daily; quetiapine, 25 mg daily; and bromazepam as needed.

On examination, the patient appeared drowsy, disoriented, and dehydrated. Physical examination revealed good hemodynamic compensation with no signs of central or peripheral congestion. Cardiac auscultation confirmed the known arrhythmia, with clear heart sounds and an abnormal diastolic murmur at Erb's point. Peripheral arterial pulses were weak. On admission, blood pressure was 80/50 mmHg, heart rate was 80/min, peripheral oxygen saturation was 95%, and the patient was apyretic. No other significant findings were reported.

Relevant laboratory tests are shown in Table 1.

**Table 1:** Laboratory tests

Laboratory tests	Values	Normal values
White blood cells	16.67 k/μL	4-10
neutrophil	87%	36-74
Red blood cells	5.24 million/μL	4,5-6
Hematocrit	40.1%	40-52
Hemoglobin	11.9 g/dL	13-17
MCV (Mean Corpuscular Volume)	76.5 fl	78-94
Platelets	136 k/Μl	150-450
INR (International Normalized Ratio)	2.75	Therapeutic range: 2-3
Partial thromboplastin time	40 seconds	25-40
Fibrinogen	429 mg/dL	180-350
AST (Aspartate Aminotransferase)	37 U/L	15-37
ALT (Alanine Aminotransferase)	17 U/L	16-63
GGT (Gamma-Glutamyl-Transferase)	41 U/L	15-85
Creatinine	2.61 mg/dL	0,67-1,11
BUN (Blood Urea Nitrogen)	81 mg/dL	7-18
Glucose	98 mg/dL	74-106
Sodium	135 mmol/L	136-145
Potassium	4.7 mmol/L	3,5-5,1
Magnesium	2.0 mg/dL	1,8-2,4
Albumin	2.4 g/dL	3,4-5

**Figure 1:**
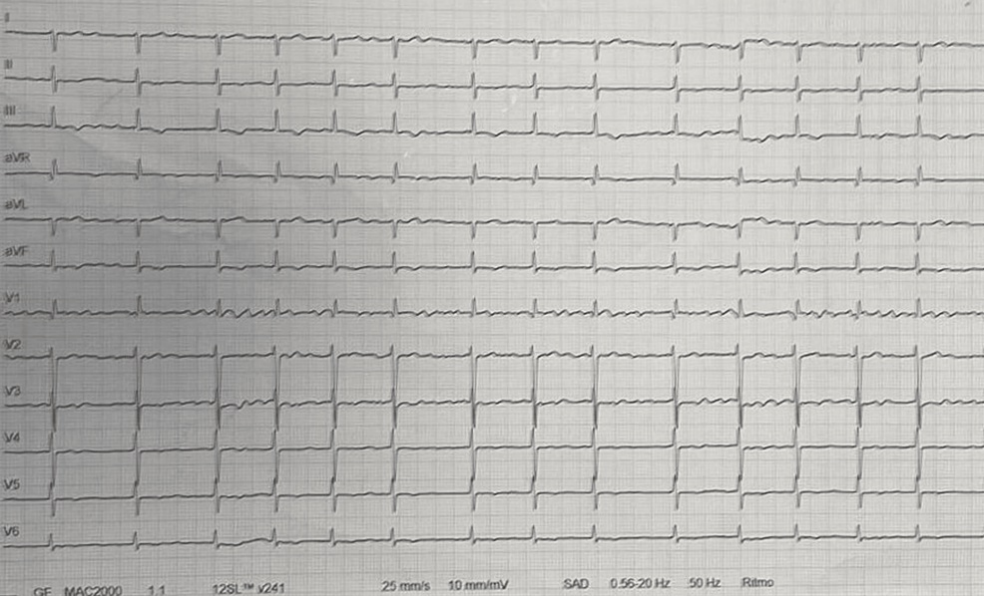
ECG

Electrocardiogram: The patient exhibited total arrhythmia due to atrial fibrillation, with a mean ventricular rate of 75/min and diffuse non-specific changes in repolarisation (Fig. 1).

**Figure 2:**
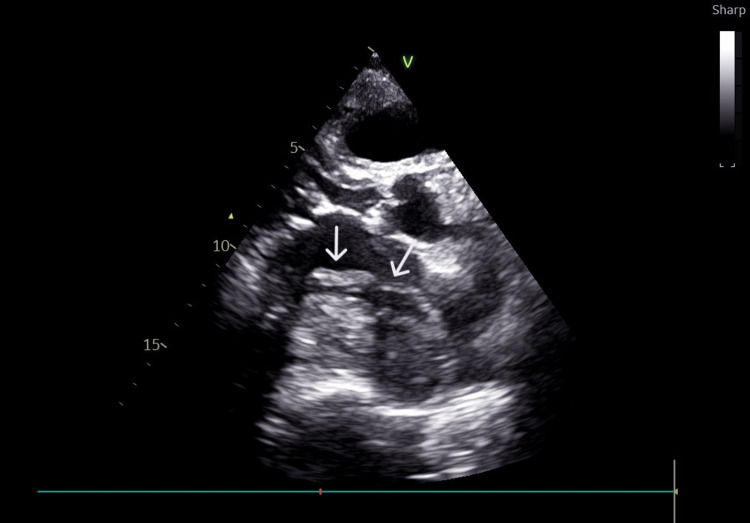
Echocardiographic image in the long-axis parasternal view showing a thrombus in the left atrium (indicated by the arrow)

Echocardiography: The aortic root, ascending aorta, and aortic arch were normal in size. The tricuspid aortic valve showed a slight reduction in opening. The left ventricle was of normal size with septal hypertrophy (interventricular septum diameter of 13 mm, posterior wall thickness of 13 mm). The ejection fraction (EF) was 60%, with no regional wall motion abnormalities. The transmitral pattern was monophasic due to atrial fibrillation. The mitral valve showed calcification, residual commissural fusion, and involvement of the subvalvular apparatus after the previous valve repair. Doppler gradients indicated severe stenosis (mean pressure gradient of 10 mmHg) and mild-to-moderate insufficiency. Severe left atrial dilatation (40 cm^2^) was observed, along with a large round mass adherent to the roof of the left atrium, which was apparently continuous with the surrounding structures. The mass had echo-lucent areas and occupied almost the entire cavity (Fig. 2, Video 1). There was a slight right atrial dilatation (25 cm^2^), while the right ventricle was of normal size (36 mm), with reduced contractility (TAPSE 11 mm). Severe pulmonary hypertension (90 mmHg), with no pericardial effusion, was observed.

**Video 1:** Long-axis parasternal echocardiography view, revealing the presence of a thrombus, mitral stenosis, and atrial dilation

**Figure 3:**
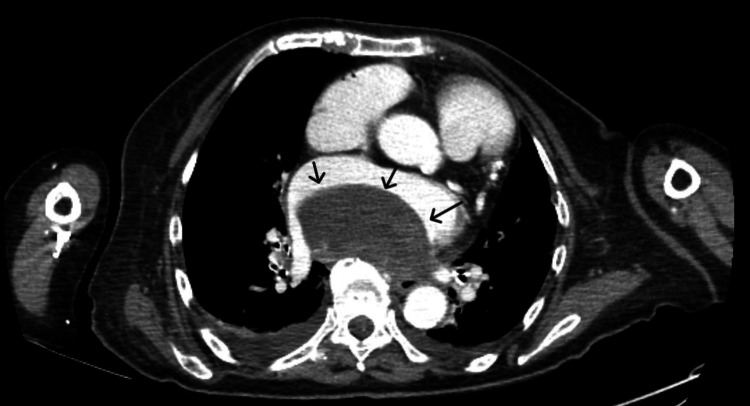
Contrast-enhanced CT image in the transverse section along the atrial plane, revealing a filling defect in the left atrium (indicated by the arrow)

Computed tomography (CT) of the chest, which was performed to exclude pulmonary inflammatory processes, confirmed the presence of a large thrombotic formation in the left atrium (Fig. 3).

The patient was subjected to subcutaneous enoxaparin therapy with a dose of 5,000 IU twice daily, together with rehydration therapy. Due to the patient's poor general condition, transoesophageal echocardiography was not performed. Over time, the patient's general clinical status gradually improved. A follow-up echocardiogram after one week revealed the persistence of the thrombotic formation in the left atrium, albeit with a slight reduction in size and evidence of remodeling (Videos 2-3).

**Video 2:** Apical four-chamber echocardiography view showing a thrombus, mitral stenosis, and atrial enlargement

**Video 3:** 3D echocardiographic reconstruction showing the atrial thrombus

After one week, warfarin therapy was started, and the patient was discharged with the recommended therapy (range of 3-3.5 for the medication).

## Discussion

This case report describes a patient with permanent atrial fibrillation, inoperable mitral stenosis, left atrial dilatation, and a significant worsening of the general conditions, which led to hospitalisation. The echocardiogram revealed a large thrombotic formation in the left atrium despite adequate anticoagulant therapy for the prevention of cardioembolism. She was on warfarin regularly, and her time to therapeutic range (TTR) for the previous 12 months was 72%. Her CHA2DS2-VASc score was high, totalling 6 points (two for age, and one for gender, heart failure, hypertension, and diabetes). She did not receive direct oral anticoagulants (DOACs) because, as we know, their indication does not include atrial fibrillation of valvular origin.

Warfarin, an oral anticoagulant, is known to inhibit vitamin K-dependent clotting factors, thereby reducing the risk of thromboembolism. The effectiveness of anticoagulation therapy in preventing thrombus formation is well-established, and therapeutic INR values within the target range are associated with a lower risk of stroke and systemic embolism in patients with atrial fibrillation [4].

This case highlights the interplay between atrial fibrillation, mitral stenosis, and left atrial dilatation as contributing factors to thrombus formation. While thrombi typically form in the appendages, it is interesting to note that, in cases of mitral stenosis, they can form directly within the main atrial chamber.

Virchow's triad teaches that thrombosis can occur when there are disruptions in blood flow, changes in the vascular endothelium, and a general state of hypercoagulability.

In patients with severe mitral stenosis, obstruction of blood flow through the stenotic valve can lead to turbulent flow. Furthermore, dilatation of the main atrial chamber and atrial appendage is associated with blood flow disturbances and stagnant blood flow, resulting in a high risk of thromboembolism [5]. This risk progressively increases with left atrial enlargement, regardless of the administration of anticoagulant therapy [3].

This observation highlights the complexity of thrombus formation in patients with multiple risk factors and seemingly contradictory findings. While the high CHA2DS2-VASc score and the presence of chronic kidney disease suggest an increased thromboembolic risk, her high TTR indicates effective anticoagulation management.

It should be noted that the development of thrombi can be influenced by various factors, including patient characteristics and underlying pathophysiological conditions. In this specific case, the presence of severe mitral stenosis and left atrial dilatation probably played a significant role in the formation of the large thrombus. These conditions are known to create a prothrombotic environment characterised by blood stasis, endothelial dysfunction, and altered flow dynamics, which collectively promote thrombus formation [6].

Some clinical cases have highlighted the risk of developing left atrial thrombosis in patients with mitral stenosis despite being treated with direct oral anticoagulants [7,8]. This case demonstrates how the failure of anticoagulation therapy in these patients can also occur with the use of properly used vitamin K inhibitors.

In our case, since VKA therapy proved to be ineffective and DOAC therapy contraindicated, we empirically decided to try to treat the patient with enoxaparin at the therapeutic dosage. At subsequent echocardiographic follow-ups, we observed an improvement understood as a reduction in thrombus size. At discharge, we again prescribed warfarin therapy to the patient, however establishing a therapeutic range of INR between 3 and 3.5. More evidence is needed to clarify the treatment of these rare cases.

Echocardiography plays a crucial role in detecting thrombi. Patients on anticoagulant therapy for the prevention of cardioembolism should have regular echocardiographic checkups to monitor for the potential development of thrombi. Early identification of thrombi can guide treatment decisions and help prevent potentially life-threatening thromboembolic events. Therefore, clinicians should remain vigilant and consider more frequent echocardiographic evaluations in patients with atrial fibrillation, mitral stenosis, and left atrial dilatation, even when they are receiving anticoagulant therapy. This approach allows for timely intervention and can help mitigate the risk of thromboembolic complications.

## Conclusions

In conclusion, regular echocardiographic evaluation is a crucial component of the management of patients with atrial fibrillation, mitral stenosis, and left atrial dilatation. It facilitates early diagnosis of thrombi despite anticoagulant therapy, guiding appropriate interventions and reducing the risk of thromboembolic events.
